# Development and validation of the dysfunctional career thoughts scale for Chinese university students

**DOI:** 10.3389/fpsyg.2025.1537321

**Published:** 2025-04-11

**Authors:** Shasha Li, Donghyuck Lee

**Affiliations:** ^1^School of Innovation and Entrepreneurship, Nanjing Vocational University of Industry Technology, Nanjing, China; ^2^Department of Education, Konkuk University, Seoul, Republic of Korea

**Keywords:** dysfunctional career thoughts, scale development, scale validation, Chinese university students, cultural differences

## Abstract

Dysfunctional career thoughts significantly impede rational career decision-making and have been widely assessed using the Career Thought Inventory (CTI). However, research suggests the CTI may not fully capture Chinese university students’ cultural uniqueness, creating a measurement gap in this population. Therefore, this study aimed to develop and validate a culturally-appropriate instrument assessing dysfunctional career thoughts among Chinese university students. From 104 preliminary items evaluated for content validity, the Dysfunctional Career Thoughts Scale (DCTS) was constructed through exploratory factor analysis with 389 students. The final 20-item instrument encompasses three dimensions: (1) Self-knowledge Uncertainty and Choice Anxiety, (2) Career Decision-Making Amotivation, and (3) Career Decision-Making Process Inefficacy. Validation with 241 additional students confirmed robust construct validity through confirmatory factor analysis. Concurrent validity was established via significant negative correlations between dysfunctional career thoughts and both career decision-making self-efficacy and vocational identity. The study findings imply that Chinese university students encounter challenges related to incomplete self-awareness, external influences, and perfectionist tendencies during the career decision-making process. This culturally-sensitive instrument offers significant advantages for academic advisors and career counselors working with Chinese university populations, providing more precise identification of intervention needs. While the DCTS demonstrates considerable theoretical and practical utility, certain limitations require further investigation in future studies.

## Introduction

Cognitive processes play a fundamental role in the quality of career decision-making (Keller et al., 1982; Sampson et al., 1996). According to Krumboltz (1994), individuals make career decisions and undertake related activities based on their self-perceptions and understanding of the professional world. He further posits that constructive and functional beliefs facilitate the achievement of career goals. The Cognitive Information Processing (CIP) theory (Peterson et al., 1991) elucidates how career-related cognitions influence decision-making processes. Research indicates that individuals with functional career thoughts exhibit decisiveness in their career choices (Peterson et al., 1996), while those with dysfunctional career thoughts encounter challenges in identifying interests and achieving career objectives (Sampson et al., 1996; Whiston and Keller, 2004).

Empirical research has demonstrated significant correlations between dysfunctional career thoughts and career-related challenges. Saunders et al.'s (2000) research on career decision-making revealed that dysfunctional career thoughts significantly predict career indecision. Similarly, Bullock-Yowell et al. (2012) established that increased dysfunctional career thoughts correlate with decreased decision satisfaction. Furthermore, studies have linked dysfunctional career thoughts to various psychological factors, including less developed personal identities (Voight, 1999), helplessness (Kilk, 1997), anger (Strausberger, 1998), depression and indecisiveness (Saunders et al., 2000), as well as frustration, guilt, and diminished interest (Corbishley and Yost, 1989; Dodge, 2001; Lewis and Gilhousen, 1981).

To assess dysfunctional career thoughts, Sampson et al. (1992) developed the Career Thoughts Inventory (CTI), grounded in CIP theory and cognitive therapy. The CTI comprises three subscales: decision-making confusion, commitment anxiety, and external conflict. Decision-making confusion reflects insufficient understanding of or difficulty initiating the decision-making process due to emotional barriers. Commitment anxiety pertains to apprehension about decision outcomes and challenges in selecting or prioritizing alternatives. External conflict indicates a tendency to avoid decision-making responsibility while struggling to reconcile information from significant others with independently acquired knowledge. The CTI effectively evaluates the extent to which dysfunctional thoughts impede career problem-solving and decision-making processes.

Extensive research on the CTI has revealed that dysfunctional career thoughts generate various impediments to career-related information processing, decision-making, and behavior. These challenges include career choice anxiety, decision postponement, excessive reliance on others, impulsive choices as stress responses, frequent job changes, reduced job search activity, limited self-awareness, biased evaluation of career alternatives, diminished motivation, choice dissatisfaction, and lack of confidence in decision-making abilities (Sampson et al., 1996). Furthermore, dysfunctional career thoughts hinder career preparation by disrupting decision-making processes and complicating the discovery of personal interests and values. These cognitive patterns manifest through verbal expression, emotional responses, and behaviors, potentially resulting in helplessness, fear, insecurity, guilt, and frustration (Corbishley and Yost, 1989).

The CIP theory was initially introduced to China by Fan (2003), with subsequent research demonstrating the effectiveness of CIP-based interventions in various contexts, including group counseling (Zang, 2006), career guidance (Zhou, 2009), and career education (Chen, 2013; Tang, 2014) among Chinese populations. Liu (2007) subsequently translated and adapted the CTI for Chinese use. This research revealed that male students exhibited higher levels of dysfunctional career thoughts, particularly in the external conflict subscale, compared to female students. Additionally, total dysfunctional career thought scores, decision-making confusion subscale scores, and decision-making styles (specifically dependent and avoidant types) emerged as significant predictors of career convictions.

Lin’s (2011) development of the Career Thoughts Inventory specifically for Chinese college students revealed significant variations in dysfunctional career thoughts across demographic and experiential factors. The findings indicated that female students, humanities majors, and those lacking internship experience demonstrated a higher propensity for negative career-related cognitions compared to their counterparts. The study also established inverse correlations between dysfunctional career thoughts and both social support and self-esteem, with self-esteem serving as a partial mediator in the relationship between social support and dysfunctional career thoughts.

Further research by Shi (2014), utilizing Liu’s (2007) adapted CTI, examined high school students’ career thoughts and revealed the influential role of family support systems. The study demonstrated that attachment style moderated the relationship between family support systems and career thoughts, with significant variations observed across gender and grade levels. Similarly, Bao’s (2016) modification of Liu’s CTI for secondary vocational high school students revealed prevalent dysfunctional career thoughts, with significant variations based on geographical origin, gender, and academic specialization.

Heine et al. (1999) posited that psychological constructs developed within Western frameworks might not adequately explain Asian behavioral patterns. The fundamental differences in Eastern and Western worldviews and cultural values significantly influence career decision-making processes (Lin, 2011; Malach-Pines and Kaspi-Baruch, 2008). Markus and Kitayama (1991) highlight that while Western cultures emphasize independence and self-actualization, Eastern cultures prioritize interdependence and social inclusion.

In the Chinese context, collective entities, particularly family units, hold paramount importance (Yang, 2004). Chinese children typically engage in collaborative problem-solving with parents, potentially resulting in limited experience with independent decision-making (Xue and Hu, 2018). The implementation of China’s single-child policy (1980–2016) has contributed to a generation of individuals who, having grown up exclusively with parents or grandparents, often exhibit heightened dependency on family members, particularly in career-related decisions (Zhang et al., 2009). Furthermore, China’s rapid globalization and digitization have significantly influenced occupational preferences among college students, particularly those born after 1990. According to Xue and Hu (2018), these “post-90s” students, raised in relatively affluent environments, demonstrate stronger preferences for economic compensation and social status. They predominantly seek employment opportunities in major urban centers and large corporations, with 59.2% prioritizing financial considerations in their career choices. This generation also exhibits a heightened focus on practical considerations and long-term career development potential, while relatively few prioritize social contribution in their career decisions.

Research on career cognition among Chinese populations underscores the necessity for assessment instruments that appropriately reflect the distinct characteristics of Chinese college students. Liu (2007) raised significant methodological concerns regarding the applicability of the CTI’s conceptualization of dysfunctional thoughts to Chinese college students’ career decision-making processes, particularly questioning whether culture-specific dysfunctional career thoughts might exist within this population. Lin’s (2011) modified CTI, which incorporated five subscales (self-understanding difficulty, decision-making startup confusion, commitment anxiety, and external environment conflict and escape), revealed structural variations in career-related cognitions between Chinese college students and their international counterparts. However, several methodological limitations warrant consideration: the escape subscale’s limited reliability due to insufficient items; restricted geographical sampling from two universities in Guangdong Province; potential conflation of undergraduate and graduate student populations without accounting for developmental differences; and item selection procedures that bypassed expert validation, potentially compromising construct validity. These limitations emphasize the critical need for developing culturally appropriate career thought assessment instruments specifically tailored to contemporary Chinese college students.

The present research endeavors to address these empirical gaps by developing and validating a psychometrically sound measure of career thoughts specifically designed for Chinese college students. The Dysfunctional Career Thoughts Scale (DCTS) for Chinese college students has been conceptualized to overcome the limitations of existing instruments while accurately capturing the cultural nuances specific to Chinese college students. Within this research framework, dysfunctional career thoughts are operationalized as the aggregate manifestation of attitudes, behaviors, beliefs, feelings, plans, and strategies related to career decision-making and problem-solving among individuals born and educated within the Chinese cultural context who are currently enrolled in Chinese higher education institutions. These dysfunctional cognitions may present through various manifestations and frequently represent inaccurate and maladaptive belief systems that impede effective career decision-making processes.

## Materials and methods

### Participants

Data collection was conducted in three distinct phases with the following participant demographics: First, for the development of the Dysfunctional Career Thoughts Scale, individual interviews and open-ended surveys were administered to both current university students in China and career counseling professionals who provide career guidance and education to university students. The expert interview group comprised 11 professionals (mean career duration: 12.9 years). The student interview cohort consisted of 15 participants (8 males, 7 females) representing diverse academic disciplines and year levels. Research indicates that in qualitative studies, data saturation can reach 85% with 12 in-depth interviews and 92% with 20 interviews (Guest et al., 2006). Thus, a recommended sample size typically ranges from 5 to 25 participants to ensure coverage of key subgroups (Creswell, 2013). The open-ended questionnaire was completed by 13 professionals (mean career duration: 7.4 years). 10 student respondents (4 males, 6 females) participated in the open-ended survey, encompassing various majors and academic years.

Second, for item selection purposes, online data were collected from 389 students (28.0% male, 72.0% female) as depicted in Table 1. In exploratory factor analysis (EFA), a sample size exceeding 300 participants is classified as excellent (Comrey and Lee, 1992). The majority of participants were aged 18 or younger (33.4%), 19 years (15.9%), and 20 years (19.5%), representing diverse geographical regions across China, including Northeast, North, East, Central, South, Southwest, and Northwest China. The institutional distribution of participants included Double First-Class universities (30.3%), standard universities (35.0%), and private universities/independent colleges (34.7%), also with participating institutions geographically dispersed throughout China. The sample encompassed students from first-year to fifth-year and spanned all major disciplinary categories: Humanities, Social Sciences, Business, Natural Sciences, Engineering, and Arts & Sports.

**Table 1:** Demographic characteristics of participants in the study.

Variable	Category	Pilot testing (*N = 389*)		Scale validation (*N = 241*)	
		*N*	%	*N*	%
Gender	Male	109	28.0	65	27.0
	Female	280	72.0	176	73.0
Age	18 or younger	130	33.4	45	18.7
	19	62	15.9	59	24.5
	20	76	19.5	51	21.2
	21	47	12.1	47	19.5
	22 or older	74	19.0	39	16.2
Region of origin	Dongbei (Northeast)	22	5.7	7	2.9
	Huadong (East)	176	45.2	70	29.0
	Huabei (North)	122	31.4	27	11.2
	Huazhong (Central)	24	6.2	9	3.7
	Huanan (South)	23	5.9	17	7.1
	Xinan (Southwest)	11	2.8	107	44.4
	Xibei (Northwest)	11	2.8	4	1.7
Region of school	Dongbei (Northeast)	40	10.3	6	2.5
	Huadong (East)	172	44.2	70	29.0
	Huabei (North)	121	31.1	31	12.9
	Huazhong (Central)	33	8.5	15	6.2
	Huanan (South)	21	5.4	10	4.1
	Xinan (Southwest)	0	0.0	108	44.8
	Xibei (Northwest)	2	0.5	1	0.4
Year in School	Freshmen	192	49.4	105	43.6
	Sophomore	48	12.3	25	10.4
	Junior	87	22.4	72	29.9
	Senior	61	15.7	38	15.8
	Fifth Year	1	0.3	1	0.4
Major	Humanities	105	27.0	57	23.7
	Social Sciences	10	2.6	51	21.2
	Business/Economics	37	9.5	29	12.0
	Science	76	19.5	29	12.0
	Engineering	97	24.9	47	19.5
	Arts/Sports	40	10.3	27	11.2
	Medicine	24	6.2	1	0.4
Work experience	Yes	200	51.4	138	57.3
	No	189	48.6	103	42.7

Third, to validate the 20 selected items, online data were collected from 241 university students (27.0% male, 73.0% female) as presented in Table 1. In confirmatory factor analysis (CFA), the sample size should generally exceed 10 times the number of items (Kline, 2016).

Similar to the item selection phase, participants represented various regions across China, with the majority (64.3%) being 20 years of age or younger representing diverse geographical regions across China, including Northeast, North, East, Central, South, Southwest, and Northwest China. The institutional composition included Double First-Class universities (18.3%), standard universities (77.6%), and private universities/independent colleges (4.1%), also with participating institutions geographically dispersed throughout China. The sample encompassed students from first-year to fifth-year and spanned all major disciplinary categories: Humanities, Social Sciences, Business, Natural Sciences, Engineering, and Arts & Sports.

## Instruments

### Dysfunctional career thoughts scale

The Dysfunctional Career Thoughts Scale (DCTS) was constructed and validated specifically for the Chinese university student population. The instrument consists of 20 items distributed across three dimensions: (1) Self-knowledge Uncertainty and Choice Anxiety (eight items), (2) Career Decision-Making Amotivation (three items), and (3) Career Decision-Making Process Inefficacy (nine items). Response options utilize a 4-point Likert-type scale ranging from 1 (Strongly Disagree) to 4 (Strongly Agree). Elevated total scores reflect higher levels of dysfunctional career cognitions. Internal consistency reliability analyses yielded a Cronbach’s alpha coefficient of 0.94 for the total scale, while subscale reliability coefficients ranged from 0.88 to 0.91.

### Career thoughts inventory

Career-related cognitions were assessed utilizing the Chinese version of Career Thoughts Inventory (CTI), adapted by Lin (2011) from Sampson et al.’s (1996) original English instrument for application within the Chinese university student population. The adapted instrument comprises 21 items distributed across five dimensions: difficulties in self-understanding (seven items), decision initiation confusion (four items), commitment-related anxiety (five items), external environmental conflicts (three items), and avoidance tendencies (two items). The instrument employs a 4-point Likert-type scale ranging from 1 (Strongly Disagree) to 4 (Strongly Agree). Higher aggregate scores indicate greater prevalence of dysfunctional career cognitions. The instrument demonstrated robust internal consistency reliability, with Cronbach’s alpha coefficients of 0.87 in the original validation study (Lin, 2011) and 0.91 in the current investigation.

### Career decision-making self-efficacy scale

Career decision-making self-efficacy was assessed using the Chinese version of the Career Decision-Making Self-Efficacy Scale (CDMSES; Peng and Long, 2001), adapted from the original instrument developed by Taylor and Betz (1983) and Betz and Taylor (1994). The instrument consists of 39 items measuring five distinct dimensions: self-evaluation (six items), occupational information acquisition (nine items), goal identification (nine items), strategic planning (eight items), and problem resolution (seven items). Responses are recorded on a 5-point Likert-type scale, ranging from 1 (not confident at all) to 5 (highly confident). Higher aggregate scores reflect elevated levels of career decision-making self-efficacy. The instrument demonstrated excellent internal consistency reliability, with Cronbach’s alpha coefficients of 0.94 in Lin’s (2011) study and 0.96 in the current investigation.

### Vocational identity scale

Vocational identity was evaluated using the Chinese version of Vocational Identity Scale (CVIS), which was adapted and validated from Holland et al.’s (1993) original Vocational Identity Scale. The Chinese adaptation, validated by Yuan (2008), consists of 18 items distributed across four dimensions: occupational interests (three items), competency assessment (five items), career decision-making processes (five items), and career goal stability (five items). The instrument employs a dichotomous response format (0 = No, 1 = Yes). Higher aggregate scores indicate stronger vocational identity, characterized by well-defined career goals, interests, personality traits, and abilities. The instrument demonstrated sound internal consistency reliability, with Cronbach’s alpha coefficients of 0.82 in Lin’s (2011) study and 0.85 in the current investigation.

## Procedure

### Item generation and content validation

The development of preliminary items for the Dysfunctional Career Thoughts Inventory (DCTS) commenced with the formulation of interview protocols and open-ended questionnaires, informed by comprehensive literature review and existing instruments. Data collection involved structured interviews and surveys administered to university students and career counseling practitioners with minimum 3 years of professional experience. The preliminary item pool was generated through systematic analysis of responses from 15 university students and 11 practitioners (interviews), supplemented by 10 university students and 13 practitioners (open-ended questionnaires).

The data collection protocols were designed to elicit comprehensive insights into Chinese university students’ career-related dysfunctional cognitions, particularly focusing on the Career Information Processing (CIP) theory’s decision-making components: communication, analysis, synthesis, valuation, and execution. The interview protocol for students incorporated queries such as “What cognitive barriers impede your self-assessment during career decision-making?” and “What thought patterns generate negative affect (e.g., anxiety, depression) during career decision-making or problem-solving processes?” Parallel inquiries were posed to practitioners regarding their observations of students’ experiences.

The open-ended questionnaire administered to students comprised eight items, including assessments of students’ occupational cognitions and perceptions of external influences (e.g., significant others, sociocultural factors, educational environment, familial background, and regulatory framework) on career choices. The practitioner questionnaire, consisting of 17 items, explored similar domains.

Initial item generation yielded 115 items, which underwent content validity assessment by five expert evaluators: two counseling psychology professors with doctoral degrees and career research expertise, one senior career counselor with over decade of experience, and two doctoral candidates with substantial career counseling research experience. The evaluation criteria encompassed appropriateness and clarity, utilizing a 5-point Likert-type scale. Items receiving mean ratings below 4 were either revised or eliminated, incorporating expert feedback. This process resulted in the elimination of 13 items and revision of 21 items, yielding 104 pilot items.

### Pilot testing and scale refinement

The psychometric properties of the 104 pilot items were examined through exploratory factor analysis (EFA) using data from 389 Chinese university students. Statistical analyses were conducted using SPSS 23.0. The item selection process followed a systematic procedure: First, item quality was assessed based on descriptive statistics, item-total correlations, and internal consistency metrics. Second, items were systematically eliminated based on established psychometric criteria: items exhibiting extreme mean values, those with standard deviations less than 0.6, items demonstrating absolute skewness or kurtosis values greater than 2 (Huck and Cormier, 1996), those with item-total correlations below 0.03 (Gable and Wolf, 1993), and items whose elimination resulted in improved scale internal consistency coefficients. Third, the data’s suitability for factor analysis was assessed using the Kaiser-Meyer-Olkin (KMO) measure of sampling adequacy and Bartlett’s test of sphericity. Fourth, an exploratory factor analysis (EFA) was conducted using maximum likelihood estimation with oblimin rotation. Fifth, correlations between the DCTS’s total score and subfactor scores were calculated, followed by an evaluation of internal consistency using Cronbach’s alpha coefficients for both the total scale and individual subfactors. This analytical process yielded a final set of 20 items, which were retained for the scale validation.

### Scale validation

The final instrument comprised 20 items derived from the pilot testing, supplemented by established measures to assess concurrent validity (Career Thoughts Inventory, Career Decision-Making Self-Efficacy Scale, and Vocational Identity Scale). Statistical analyses were conducted using SPSS 23.0 and MPLUS 7 software packages on data obtained from a sample of 241 undergraduate students. The psychometric evaluation proceeded in three phases: First, internal consistency reliability was assessed via Cronbach’s alpha coefficients for all measures. Subsequently, construct validity was examined through confirmatory factor analysis (CFA). The structural equation model’s goodness-of-fit was evaluated using multiple indices, including chi-square statistics, comparative fit index (CFI), Tucker-Lewis index (TLI), and root mean square error of approximation (RMSEA). While the chi-square statistic has traditionally been employed in model fit evaluation, its sensitivity to sample size and tendency toward Type I error necessitated its consideration primarily as a supplementary indicator. Therefore, primary emphasis was placed on the CFI and TLI, established relative fit indices for which values ≥0.90 indicate satisfactory model fit (Hu and Bentler, 1999). The RMSEA served as the principal absolute fit index, with interpretive thresholds as follows: values <0.05 indicating close fit, values <0.08 suggesting reasonable fit, and values >0.10 representing unacceptable fit (Browne and Cudeck, 1993). Moreover, in confirmatory factor analysis (CFA), modification indices (MI) identify potential model misspecifications by estimating the expected decrease in the chi-square statistic (*χ*^2^) if constraints on specific model parameters are relaxed (Bollen, 1989). Following methodological conventions (Kline, 2016), MI values ≥10 suggest that the constraints on the parameter may lead to significant model misfit. Finally, concurrent validity was established by examining the correlational patterns among career thoughts, career decision-making self-efficacy, and vocational identity constructs.

## Results

### Pilot testing and scale refinement

The preliminary scale refinement involved systematic psychometric evaluation. Items exhibiting restricted variance (SD ≤ 0.60) were eliminated from the initial 104-item pool, resulting in the removal of six items. Analysis of distributional characteristics demonstrated that all retained items met normality assumptions as assessed through skewness and kurtosis indices (skewness: −0.87 ~ 0.60, kurtosis: −0.85 ~ 1.67). All items’ skewness and kurtosis values should fall within ±2 to satisfy an acceptable range of item distribution (Huck and Cormier, 1996). Further item analysis utilizing corrected item-total correlations led to the elimination of one additional item that demonstrated inadequate discrimination (*r* < 0.30).

An exploratory factor analysis (EFA) was conducted utilizing maximum likelihood estimation with oblimin rotation. The sampling adequacy was assessed via the Kaiser-Meyer-Olkin (KMO) measure, yielding a coefficient of 0.96, while Bartlett’s test of sphericity produced a significant result (*χ^2^* = 24648.76, *df* = 4,656, *p* < 0.001), indicating the data’s suitability for factor analysis. Factor extraction was informed by multiple criteria, including eigenvalues, scree plot examination, and theoretical interpretability of factor structures, ultimately supporting a three-factor solution. Item retention was determined through established psychometric criteria: communalities exceeding 0.30, primary factor loadings above 0.50, and absence of cross-loadings exceeding 0.30. Through successive iterations of factor analysis, 20 items were retained across three factors. The resultant factor structure accounted for 63.44% of the total variance, with Factor 1 accounting for 46.02%, Factor 2 for 11.00%, and Factor 3 for 6.41%. This cumulative explained variance exceeded the conventional threshold of 40%, supporting the adequacy of the three-factor model. Based on conceptual analysis of item content, the factors were designated as: (1) Self-knowledge Uncertainty and Choice Anxiety, (2) Career Decision-Making Amotivation, and (3) Career Decision-Making Process Inefficacy. The final factor structure and corresponding item loadings are presented in Table 2.

**Table 2:** Final items of dysfunctional career thoughts inventory and results of exploratory factor analysis (Beaton et al., 2000).

Factor		Items	Factor Loadings			Communality
			1	2	3	
Self-knowledge uncertainty and choice anxiety	7	I’m afraid I will not find an occupation that suits my ability.	0.91	−0.00	−0.13	0.67
	8	I’m worried that I will not be able to perform in my chosen occupation due to lack of ability.	0.82	0.00	−0.06	0.61
	10	I am worried about not being able to choose an occupation that is right for me.	0.71	−0.02	0.15	0.68
	6	I am not sure what I am good at.	0.70	0.06	0.05	0.58
	9	I do not know which occupation is right for me.	0.69	0.02	0.13	0.64
	11	I do not have enough or deeper understanding of myself.	0.68	0.01	−0.01	0.46
	12	I do not have the means to get an overall and deeper understanding of myself.	0.65	0.00	0.08	0.51
	13	I do not know how much what I learned in university can help me choose my occupation.	0.51	0.01	0.11	0.36
Career Decision-Making Amotivation	48	I do not have to consider choosing my occupation because no one has ever pressed me to choose one.	−0.01	0.88	0.00	0.78
	47	I do not have to consider choosing my occupation because those around me have not started choosing their occupations.	−0.00	0.85	0.03	0.74
	44	I do not have to consider choosing my occupation because getting a job is still far from now.	0.04	0.77	−0.01	0.61
Career Decision-Making Process Inefficacy	80	I’m unlikely to choose one out of the occupations I could consider.	−0.09	−0.03	0.83	0.56
	76	I am so confused now that I cannot choose a job.	−0.09	0.09	0.79	0.60
	88	I do not know how to prepare to have an occupation.	0.01	0.03	0.78	0.64
	98	Choosing an occupation is such a complex task that I do not know which stage I’m in now.	0.10	0.01	0.73	0.66
	100	I’m frustrated and troubled because I cannot choose a satisfactory occupation.	0.14	−0.11	0.67	0.55
	96	I get anxious whenever I think about entering society alone and working.	0.04	−0.02	0.64	0.44
	91	I’ve tried many times, but I could not choose a satisfactory occupation.	0.00	0.11	0.64	0.48
	83	I do not know which occupation I like because of a lack of experience.	0.13	0.04	0.61	0.53
	56	I wish there was a simple and quick way to choose an occupation.	0.07	−0.02	0.59	0.40

Three factors were extracted via exploratory factor analysis (EFA), with gray-shaded regions in the factor loading matrix visually demarcating the structural boundaries of each factor, thereby illustrating their categorical distinctiveness.

Examination of the correlation matrix (Table 3) revealed substantial positive associations between total and subfactor scores (*r* = 0.50–0.93), while interfactor correlations exhibited moderate magnitudes (*r* = 0.26–0.71). This pattern of correlations supports the factorial distinctiveness of the subscales while maintaining conceptual coherence, suggesting that the subfactors effectively capture discrete yet related dimensions of career-related cognitions in the Chinese university student population. Psychometric evaluation of internal consistency yielded robust reliability coefficients, with the total scale demonstrating excellent internal consistency (*α* = 0.94) and subfactors showing strong reliability indices (*α* = 0.88–0.91), thus establishing the measure’s psychometric integrity.

**Table 3:** Correlation coefficients and reliability coefficients of DCTS factors (*N* = 389).

Factor	Factor 1	Factor 2	Factor 3	Cronbach’s α
Factor 1	—			0.91
Factor 2	0.26^*^	—		0.88
Factor 3	0.71^*^	0.39^*^	—	0.91
Total	0.89^*^	0.50^*^	0.93^*^	0.94

Factor 1: Self-knowledge uncertainty and choice anxiety; Factor 2: Career decision-making amotivation; Factor 3: Career decision-making process inefficacy. *p < 0.01.

### Scale validation

Initial model estimation yielded the following fit indices: *χ^2^* (167) = 417.72, CFI = 0.89, TLI = 0.87, and RMSEA = 0.06. Examination of modification indices revealed substantial residual covariation between Items 7 and 8 (MI = 39.54) and Items 11 and 12 (MI = 33.03), suggesting shared method variance beyond that accounted for by the hypothesized factors. Model respecification incorporating these residual correlations resulted in improved fit: *χ^2^* (165) = 346.95, CFI = 0.92, TLI = 0.91, and RMSEA = 0.06, indicating satisfactory model-data correspondence.

Concurrent validity was evaluated through correlation analyses between the DCTS and established criterion measures (Table 4). Analyses encompassed both total scores and subfactor scores for the DCTS in relation to the Career Thoughts Inventory (CTI), Career Decision-Making Self-Efficacy Scale (CDMSES), and Vocational Identity Scale (VIS). Detailed correlation matrices are presented in Table 4.

**Table 4:** Correlations between DCTS, CTI, CDMSE, and VI (*N* = 241).

Scale	Factor	Total	Self-knowledge Uncertainty and Choice Anxiety	Career Decision-Making Amotivation	Career Decision-Making Process Inefficacy
Chinese version of career thoughts inventory	Total	0.85	0.75	0.32	0.79
	Self-knowing difficulty	0.74	0.71	0.29	0.63
	Decision-making startup confusion	0.75	0.69	0.24	0.66
	Commitment anxiety	0.67	0.53	0.20	0.70
	External environment conflict	0.60	0.52	0.13	0.60
	Escape	0.53	0.40	0.30	0.53
Career decision-making self-efficacy	Total	−0.62	−0.59	−0.24	−0.54
	Self-Evaluation	−0.62	−0.59	−0.20	−0.54
	Occupational information gathering	−0.53	−0.50	−0.22	−0.46
	Goal Selection	−0.63	−0.58	−0.22	−0.56
	Planning	−0.56	−0.53	−0.25	−0.47
	Provlem Solving	−0.54	−0.50	−0.23	−0.47
Vocational identity	Total	−0.68	−0.64	−0.21	−0.61
	Vocational interest	−0.53	−0.51	−0.18	−0.46
	Ability assessment	−0.62	−0.59	−0.22	−0.55
	Career decision	−0.56	−0.51	−0.21	−0.51
	Stability of occupational goal	−0.46	−0.43	−0.04	−0.44

*p < 0.01.

The DCTS demonstrated significant positive correlations with all CTI dimensions (*r* = 0.13–0.85, *p* < 0.01), suggesting convergent validity. Consistent with theoretical expectations, significant negative correlations emerged between the DCTS and CDMSES (*r* = −0.63–-0.20, *p* < 0.01). Similarly, the DCTS exhibited significant negative correlations with most VIS dimensions (*r* = −0.68–-0.18, *p* < 0.01), with the sole exception being the correlation between career decision-making amotivation and stability of occupational goals. These systematic patterns of associations provide robust evidence for the concurrent validity of the DCTS.

## Discussion

The primary objective of this study was to develop a scale to measure dysfunctional career thoughts among Chinese university students. The research was conducted in three phases: First, individual interviews and open-ended surveys were administered to Chinese university students and career counseling professionals who provide career guidance and educational services in university settings to develop items measuring dysfunctional career thoughts. Second, to select appropriate items, a preliminary assessment comprising these items was administered to 389 Chinese university students, resulting in the selection of 20 items through data analysis. Third, to validate the selected items, the 20-item scale and other assessments were administered to an additional 241 Chinese university students to verify construct and concurrent validity. This process resulted in the development of the Dysfunctional Career Thoughts Scale (DCTS), consisting of 20 items across three factors.

The DCTS comprises the following subscales: The first factor, “Self-Knowledge Uncertainty and Choice Anxiety (UCA)” (8 items), measures anxiety about making appropriate choices, apprehension about future workplace life, and fear of incorrect decisions due to insufficient self-understanding. The second factor, “Career Decision-Making Amotivation (CDA)” (3 items), assesses insufficient recognition of the necessity for career decision-making. The third factor, “Career Decision-Making Process Inefficacy (CDI)” (9 items), evaluates insufficient understanding of and confidence in the career decision-making process. Among these three subscales, the UCA factor accounted for the highest proportion of total explained variance, followed by the CDA factor, while the CDI factor contributed the least variance. These findings suggest that the primary factors impeding career decision-making among Chinese university students are insufficient self-understanding and anxiety about career choices, followed by lack of motivation and confidence in the career decision-making process.

These findings align with previous research indicating that Chinese university students exhibit fragmented self-conceptualization (Li, 2017). Yu and Ma (2016) posited that university career guidance centers predominantly emphasize administrative processes and policy dissemination while neglecting crucial services such as vocational assessment and individualized career exploration. Furthermore, they noted that insufficient expertise among career counselors regarding self-exploration methodologies and decision-making processes may diminish students’ self-efficacy in career planning.

The research also revealed that Chinese undergraduates often perceive career-related anxiety and uncertainty as pathological states rather than natural components of the decision-making process. This perspective extends to post-graduation employment anxiety. These findings corroborate existing literature: 64% of university students report employment-related anxiety, with approximately 40% experiencing moderate to severe symptoms (Jiao, 2015). Additional studies indicate that career anxiety stems from inadequate psychological preparation, diminished confidence, and limited understanding of both employment landscapes and self-awareness (Cai and Li, 2007). Zhang et al. (2018) demonstrated that this anxiety, rooted in insufficient understanding of self, occupational environments, and decision-making processes, creates a recursive cycle that further impairs career decision-making capability.

Moreover, the research indicates that Chinese undergraduates typically initiate career planning in response to external pressures rather than intrinsic motivation, potentially compromising decision-making effectiveness. This observation aligns with Guo’s (2017) findings regarding Chinese students’ diminished initiative and its correlation with reduced independence, critical thinking, and problem-solving capabilities. Li (2017) further noted a significant deficit in students’ recognition of career planning’s importance. This passive approach may exacerbate anxiety, as Song (2015) demonstrated an inverse relationship between initiative and employment-related anxiety. The tendency to postpone career decisions, often justified by an assumption that opportunities will materialize spontaneously (Fu et al., 2014), can adversely affect subsequent career outcomes (Jiang, 2014).

### Comparison with the CTI

When comparing these results with Sampson et al.'s (1996) Career Thoughts Inventory (CTI), several observations emerge. The DCTS’s first factor (Self-Knowledge Uncertainty and Choice Anxiety) and the third factor (Career Decision-Making Process Inefficacy) share conceptual similarities with CTI’s Decision-Making Confusion (DMC) and Commitment Anxiety (CA) factors. However, CTI’s External Conflict (EC) factor is absent in the DCTS, while DCTS’s DCA factor is not present in the CTI. The absence of the CTI’s EC factor in the DCTS can be speculated through Lee et al.’s (2016) comparative study of CTI factor structures among Korean and American college students. Their research revealed that Korean students did not perceive the EC as an independent factor but rather as integrated with DMC factor. This suggests that external conflicts might be so fundamentally embedded in East Asian career decision-making processes that they permeate all factors rather than existing as a distinct dimension.

The DCA factor in career decision-making, unique to the DCTS, appears to reflect distinctive characteristics of Chinese university students. Research indicates that Chinese students often pursue perfectionism in career choices, desiring complete self-understanding and precise career information. Zeng (2009) noted that Chinese university students demonstrate relatively high desires for perfect self-knowledge and career fit. Yang (2015) further demonstrated the significant impact of negative perfectionism on career indecision among Chinese students. He (2018) pointed out that Chinese students born after 1995 often struggle with career decisions due to unrealistic ideals.

This perfectionist tendency appears connected to broader East Asian cultural characteristics, particularly collectivism and emphasis on group harmony. Chinese students’ sensitivity to parental evaluation (Heine, 2001; Markus and Kitayama, 1991; Oishi and Diener, 2001) and East Asian parents’ typically high expectations contribute to socially prescribed perfectionism (Castro and Rice, 2003; Flett et al., 1995; Park and Kim, 2006). Choi’s (2020) research on Korean students’ dysfunctional career thoughts revealed that fear of disappointing parental expectations often leads to decreased career preparation motivation. These findings suggest that Chinese collectivist cultural values and associated high levels of socially prescribed perfectionism significantly influence career decision-making processes, potentially contributing to motivational deficits in career preparation.

### Implications and limitation

This research makes several valuable contributions to the field of career development and assessment. Primarily, it introduces a culturally calibrated career cognition assessment instrument specifically designed for Chinese undergraduate students, addressing notable limitations in both the original Career Thoughts Inventory (CTI) and its Chinese translation. The study’s analysis of factor structure and item composition reveals distinct patterns of dysfunctional career thoughts among Chinese university students compared to their American counterparts, highlighting the inadequacy of simply using translated assessment tools.

This finding underscores a critical consideration for practitioners: assessment instruments must be culturally appropriate for their intended population. The use of non-culturally adapted tools may lead to imprecise or potentially misleading results. Therefore, practitioners should prioritize utilizing either culturally adapted assessments or instruments developed specifically for the indigenous population under consideration.

Furthermore, the research identifies specific cognitive patterns that impede career decision-making among Chinese undergraduates, providing career counselors with actionable insights for intervention development. The findings illuminate three key impediments: a distinctive lack of motivation for career decision-making, a strong correlation between insufficient self-knowledge and choice anxiety, and a notable deficit in confidence regarding the career decision-making process.

These insights enable career counselors to develop more targeted and effective interventions. Following the framework established by Sampson et al. (1996), counselors can employ cognitive reframing techniques and implement the identify-challenge-alter and act approach to assist Chinese undergraduates in transforming dysfunctional career thoughts into productive decision-making strategies. This methodological approach allows for precise identification of problematic thought patterns and facilitates the development of tailored intervention strategies.

Several limitations warrant consideration. First, the study’s sample size and institutional representation may limit generalizability given China’s vast university population. Gender distribution within the sample was suboptimal. Future research should incorporate a broader range of institutions and achieve better gender balance. Second, demographic variables’ influence on career cognitions requires further investigation. The absence of established norms for the DCTS necessitates additional standardization studies. Third, while this instrument targets university students, future research should consider developing similar measures for other career decision-making populations. Finally, it is noteworthy that while the DCTS utilized a 4-point Likert scale, the analysis did not account for the ordinal nature of Likert items (Zumbo et al., 2007). For future investigations, researchers should implement other statistical methods, including ordinal alpha in place of Cronbach’s alpha, Diagonally Weighted Least Squares estimation rather than maximum likelihood estimation, and polychoric correlations instead of Pearson correlations.

This research developed the Dysfunctional Career Thoughts Scale (DCTS) and validated it as a psychometrically sound measure instrument. This work addresses empirical gaps in the field of career thoughts research among Chinese university students, overcomes the limitations of existing measurement tools, and accurately captures culturally nuanced career cognitive characteristics specific to the Chinese cultural context. Future research could leverage the psychometric instrument developed in this study to formulate targeted intervention strategies that facilitate the transformation of dysfunctional career thoughts into functional career thoughts among Chinese university students. Additionally, subsequent investigations should extend this line of inquiry to other career decision-making populations.

## Data Availability

The original contributions presented in the study are included in the article/supplementary material, further inquiries can be directed to the corresponding author/s.
